# Normal DNA Methylation Dynamics in DICER1-Deficient Mouse Embryonic Stem Cells

**DOI:** 10.1371/journal.pgen.1002919

**Published:** 2012-09-06

**Authors:** Jonathan Ip, Paul Canham, K. H. Andy Choo, Yoshimi Inaba, Shelley A. Jacobs, Paul Kalitsis, Deidre M. Mattiske, Jane Ng, Richard Saffery, Nicholas C. Wong, Lee H. Wong, Jeffrey R. Mann

**Affiliations:** 1 Theme of Genetic Disorders, Murdoch Childrens Research Institute, The Royal Children's Hospital, Parkville, Victoria, Australia; 2 Department of Zoology, The University of Melbourne, Melbourne, Victoria, Australia; 3 Theme of Cell Biology, Development, and Disease, Murdoch Childrens Research Institute, The Royal Children's Hospital, Parkville, Victoria, Australia; 4 Department of Biochemistry and Molecular Biology, Monash University, Melbourne, Victoria, Australia; Queensland Institute of Medical Research, Australia

## Abstract

Reduced DNA methylation has been reported in DICER1-deficient mouse ES cells. Reductions seen at pericentric satellite repeats have suggested that siRNAs are required for the proper assembly of heterochromatin. More recent studies have postulated that the reduced methylation is an indirect effect: the loss of *Mir290* cluster miRNAs leads to upregulation of the transcriptional repressor RBL2 that targets the downregulation of DNA methyltransferase (*Dnmt*) genes. However, the observations have been inconsistent. We surmised that the inconsistency could be related to cell line “age,” given that DNA methylation is lost progressively with passage in DNMT-deficient ES cells. We therefore subjected *Dicer1* ^−/−^ ES cells to two experimental regimes to rigorously test the level of functional DNMT activity. First, we cultured them for a prolonged period. If DNMT activity was reduced, further losses of methylation would occur. Second, we measured their DNMT activity in a rebound DNA methylation assay: DNA methylation was stripped from Cre/*loxP* conditionally mutant *Dicer1* ES cells using a shRNA targeting *Dnmt1* mRNA. Cre expression then converted these cells to *Dicer1* ^−/−^, allowing for DNMT1 recovery and forcing the cells to remethylate in the absence of RNAi. In both cases, we found functional DNMT activity to be normal. Finally, we also show that the level of RBL2 protein is not at excess levels in *Dicer1* ^−/−^ ES cells as has been assumed. These studies reveal that reduced functional DNMT activity is not a salient feature of DICER1-deficient ES cells. We suggest that the reduced DNA methylation sometimes observed in these cells could be due to stochastic alterations in DNA methylation patterns that could offer growth or survival advantages in culture, or to the dysregulation of pathways acting in opposition to the DNMT pathway.

## Author Summary

In mammalian cells, DNA methylation is required for the maintenance of genome stability. Recent studies have shown that the genome-wide levels of DNA methylation can be reduced in DICER1-deficient mouse embryonic stem (ES) cells, suggesting that the activity of DNA methylating enzymes (DNMTs) may be regulated by small RNA molecules. The enzyme DICER1 catalyses the production of these small RNAs that serve as sequence-specific guides for modifying chromatin or transcription. However, these observations of defective DNA methylation have been inconsistent. We surmised that this inconsistency could be due to cell line “age,” because it can take many cell divisions before reduced DNMT activity may result in loss of DNA methylation. To test this idea, we rigorously assayed the functional level of DNMT activity in DICER1-deficient ES cells. First, we tested their ability to maintain DNA methylation over prolonged culture. Second, we tested their ability to rebound in DNA methylation after first stripping it from the genome. In both cases functional DNMT activity was entirely normal. We suggest that losses of DNA methylation sometimes seen in DICER1-deficient ES cells is stochastic and could involve cell line adaptation.

## Introduction

Most CpG dinucleotides in the mammalian genome are methylated [1], much of this being present at localized centric (minor satellite, MinS) and pericentric (major satellite, MajS) repeats, and dispersed repeats or transposable elements (TEs). Here DNA methylation plays a role in maintaining genome stability, being required for chromosome (Chr) stability [2], [3] and the silencing of TEs [4], [5]. At single copy sequences, the stabilization of transcriptional repression through promoter methylation is a common theme, although other functions for DNA methylation within the gene body and at regulatory regions are apparent [6]. For example, DNA methylation is central to the establishment and maintenance of the parental-specific expression of imprinted genes [7], and for the stabilization of X Chr inactivation [8]. Given the wide-ranging and essential functions of DNA methylation in mammalian cells, it is important to identify mechanisms that assist in its establishment and propagation.

A number of studies have reported a disruption in DNA methylation at repetitive sequences in mouse embryonic stem (ES) cells lacking activity of the key RNAi enzyme DICER1 (Dicer1, Dcr-1 homolog (Drosophila)). Two different mechanisms have been suggested for these methylation defects. In the first mechanism, it was suggested that reduced DNA methylation at MajS and MinS repeats is linked with a failure in RNAi-mediated heterochromatinization [9]. This mechanism has been extensively studied in regard to the pericentric region in fission yeast (*Schizosaccharomyces pombe*). Transcripts from this region are processed into siRNAs by Dicer, which then guide the Clr4 histone methyltransferase back to the repeats to catalyse the formation of histone H3 lysine 9 trimethylation (H3K9me3). This post-translational modification (PTM) is an essential waypoint in heterochromatin induction and recruits heterochromatin-inducing proteins such as Swi6 [10], [11]. The latter part of this pathway in fission yeast is conserved in mammals. Pericentric H3K9me3 is catalysed by SUV39H1 (suppressor of variegation 3–9 homolog 1 (Drosophila)) and SUV39H2, the mammalian homologues of Clr4. This PTM then recruits the heterochromatin-inducing CBX proteins, the mammalian homologues of Swi6. In addition, H3K9me3 recruits de novo DNA methyltransferase (DNMT) activity and DNA methylation, thereby adding an additional repressive layer [2], [3]. If pericentric DNA methylation is indeed defective in DICER1-deficient ES cells [9], then this would point to earlier parts of the pathway also being conserved, that is, mammalian pericentric H3K9me3 could also be guided by siRNAs that are processed from MajS transcripts by DICER1. However, defective DNA methylation at MajS repeats in ES cells has not been consistently observed. While one study observed losses [12], three others did not [13]–[15].

The second mechanism attributes losses of DNA methylation in DICER1-deficient ES cells, or their immediate differentiated derivatives, to an indirect effect of the loss of DICER1 pre-miRNA processing. More specifically, it has been proposed that *Mir290* (microRNA 290) cluster miRNA deficiency results in an upregulation of a target transcript encoding the transcriptional suppressor RBL2 (retinoblastoma-like 2), with increased RBL2 in turn suppressing *Dnmt* gene transcription and lowered DNMT protein. Sequences reduced in DNA methylation were the promoters of pluripotency genes on ES cell differentiation [15], the whole genome [12], and the *Xist* (inactive X specific transcripts) promoter [16]. However, the effects observed on *Dnmt* expression and DNA methylation were again inconsistent. For example, a generalized reduction in genomic DNA was reported, including a loss of DNMT1 activity [12], that was not observed in another study [15]. Also, the degree of demethylation at the *Xist* promoter varied between sublines [16]. It should be noted that the relative level of RBL2 protein in DICER1-deficient ES cells was not measured in immunoblots [12], [15]. A summary of the various findings in DICER1-deficient ES cells published to date is provided (Table S1).

While RNAi plays important roles in establishing epigenetic states in fission yeast and in plants, the latter possessing a robust RNAi-mediated DNA methylation mechanism [17], it remains unclear that RNAi might play similar roles in mammalian cells. Because of the broad implications for our understanding of the maintenance of genome stability, differentiation, and X Chr inactivation, it is important to resolve if there does exist direct or indirect mechanistic links between RNAi and DNA methylation in ES cells or other mammalian cell types. We therefore decided to reinvestigate the effects of ablating *Dicer1* on DNA methylation at various sequences in ES cells. We considered that, if DNMT activity was critically impaired in *Dicer1* ^−/−^ ES cells, then the inconsistent losses of DNA methylation across the various studies could be related to the length of time the lines used had previously spent in culture. For example, impaired maintenance DNMT activity would lead to a rapid loss of global methylation with passage, while impaired de novo DNMT activity would lead to a gradual global loss. To rigorously test this possibility, we subjected *Dicer1* ^−/−^ ES cells to two types of experimental manipulation designed to amplify the effect of reduced DNMT activity. These were (i) a prolonged culture regime, and (ii) a test of their ability to ‘rebound’ in DNA methylation after experimentally stripping them of DNA methylation. In neither case did we obtain evidence for defective functional DNMT activity. We conclude that the reductions of DNMT protein levels that can be observed in DICER1-deficient ES cells are generally insufficient to account for losses in DNA methylation. We propose that the losses of DNA methylation sometimes observed could involve an adaptive response of the cells to the lack of si- and miRNAs, or even the upregulation of pathways that work in opposition to the DNMT machinery.

## Results

### Effect of DICER1-deficiency on *Dnmt* and *Rbl2* mRNA and encoded protein levels

A euploid *Dicer1* ^c/−^ (c, Cre/*loxP* conditional mutant allele; -, mutant allele) mouse ES cell line of XY sex Chr constitution was derived. The *Dicer1* mutation [18] involves deletion of the terminal three exons encoding 76 of 213 aa of the second RNase III catalytic domain and all 57 aa of the dsRNA binding domain. Also, the translation stop codon and poly(A) signal sequences are deleted, these being generally required for transcript stability and nuclear export, respectively. The *Dicer1* ^c/−^ ES cells were transiently transfected with a Cre expression cassette to obtain *Dicer1* ^−/−^ clones. These were distinguishable at picking by their small size, with genotypes confirmed by Southern blot. Picked clones were expanded and DNA and RNA harvested at passage 5.

**Figure 1:**
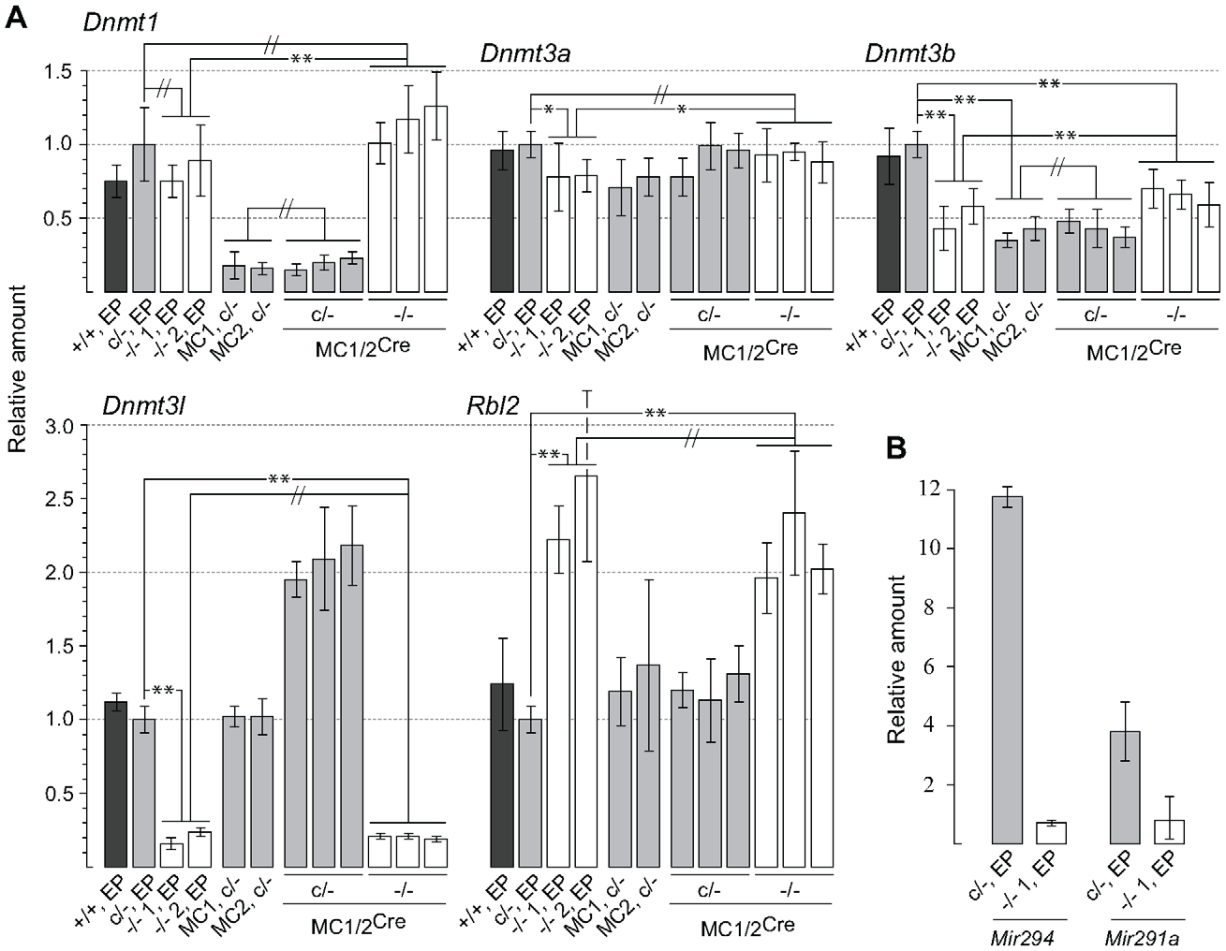
qPCR assays. (A) Values obtained for the parental *Dicer1* ^c/−^ line were used to calibrate all other values, and are set at 1.0. The *y* axis is a linear scale. Bars are mean ± s.d. Probabilities obtained from Student's *t*-test, 2-tail, type 2: *P*<0.05 (*), *P*<0.01 (**), not significantly different (//). Bar 1; *Dicer1* ^+/+^ cell line 2A at early passage (EP). Bar 2; parental *Dicer1* ^c/−^ EP line. Bars 3 and 4; Two independent *Dicer1* ^−/−^ EP lines derived from Cre transfection of the parental *Dicer1* ^c/−^ EP line. Bars 5 and 6; Two independent sublines (MC1 and MC2) derived from concatemeric targeting at *Hprt* with the *Dnmt1* shRNA. Bars 7–9 and 10–12; three sublines derived from exposure of MC1 (two sublines) and MC2 (one subline) to Cre (MC1/2) that did not excise (c/−) and did excise (−/−) the floxed *Dicer1* sequence, respectively. (B) Measurements were of the parental *Dicer1* ^c/−^ line and one *Dicer1* ^−/−^ subline. The *y* axis is a linear scale. Values are normalized to those obtained for *Rps7*, which are set at 1.0. Other details are contained in the Materials and Methods section.

qPCR assays showed that, in the two independently derived *Dicer1* ^−/−^ lines at early passage (EP), the amount of *Dnmt1* (DNA methyltransferase (cytosine-5) 1) mRNA was unchanged relative to the parental *Dicer1* ^c/−^ EP cell line (Figure 1A). By contrast, the amounts of *Dnmt3a*, *Dnmt3b* (DNA methyltransferase 3A and -B) and *Dnmt3l* (DNA (cytosine-5-)-methyltransferase 3-like) mRNAs were significantly reduced to ∼75%, 50% and 20% respectively, while the amount of *Rbl2* mRNA was increased to ∼200%. *Mir290* cluster miRNAs were essentially undetectable in our *Dicer1* ^−/−^ lines (Figure 1B). Overall, these findings indicate that, in terms of the mRNA levels for these genes, our *Dicer1* ^−/−^ EP ES cell lines were similar to those previously described [12], [15], [16].

**Figure 2:**
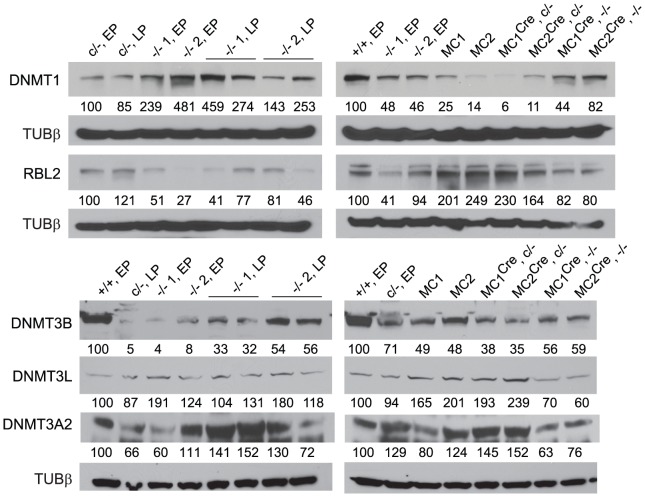
Immunoblots. Values below each band represent the densitometry reading taken for that band. These were normalized to the reading taken for lane 1, which was for the WT *Dicer1* ^+/+^ EP cell line (+/+, EP), or the parental *Dicer1* ^c/−^ EP cell line (c/−, EP). The loading was consistent throughout as shown by the detection of Tubulin β (TUBβ).

In the *Dicer1* ^−/−^ EP cell lines, immunoblots (Figure 2) showed that there was no deficiency in DNMT1 relative to the parental *Dicer1* ^c/−^ EP line (left blot). Indeed, DNMT1 levels were elevated. The levels of DNMT3A2 in the *Dicer1* ^−/−^ EP lines were on average ∼85% of that present in the *Dicer1* ^+/+^ EP line (left blot). Surprisingly, the relative levels of the other proteins assayed were very different to their corresponding mRNAs (Figure 2). In the *Dicer1* ^−/−^ EP lines, DNMT3B levels were less than 10% that in the *Dicer1* ^+/+^ EP line (left blot). However, they were probably not as deficient relative to the parental *Dicer1* ^c/−^ EP line, as this line had ∼70% the amount of DNMTB in comparison to the *Dicer1* ^+/+^ EP line (right blot). Despite the low amount of *Dnmt3l* mRNA in *Dicer1* ^−/−^ EP lines, DNMT3L protein content in these lines was similar if not higher relative to the *Dicer1* ^+/+^ EP line (left blot). Significantly, the amount of RBL2, for which elevated levels have been proposed to cause repression of *Dnmt* genes and consequent demethylation in ES cells, was lower in the *Dicer*1^−/−^ EP cells compared to the parental *Dicer1* ^c/−^ and *Dicer1* ^+/+^ EP lines (left and right blots).

### DICER1-deficient ES cells retain DNA methylation over extended passage

The large majority of CpG dyads in the mammalian genome are symmetrically methylated. The stability of this high degree of DNA methylation, at least in ES cells, relies on the presence of de novo and maintenance DNMT activity. De novo activity is provided largely by DNMT3A and DNMTB working with the cofactor DNMT3L. These enzymes are able to hemimethylate unmethylated CpG dyads, or fully methylate hemimethylated dyads. However, the system is relatively inefficient, that is, the proportion of sites that can be de novo methylated per cell division is low. Maintenance activity is provided largely by DNMT1. With high efficiency, this enzyme fully methylates hemimethylated dyads produced at DNA replication. Loss of the de novo (DNMTs 3A, 3B and 3L) or maintenance (DNMT1) system results in the progressive loss of DNA methylation at virtually all sequences. On ablation of *Dnmt1*, global loss occurs over 5 passages (25 cell divisions), although a background of methylation is retained indefinitely due to continuing de novo DNMT activity. This ongoing de novo activity is crucial for the remethylation of the genome should DNMT1 activity be reintroduced, as DNMT1 alone has negligible de novo activity in ES cells. Ablating *Dnmt3a* and *Dnmt3b*, or *Dnmt3l*, results in a much slower loss of DNA methylation than does ablation of *Dnmt1*. Unmethylated dyads produced stochastically can no longer be remethylated with any efficiency so they gradually accumulate. In *Dnmt3a*-*Dnmt3b* double-mutant ES cells, virtually all methylation is eventually lost [1], [19]–[27].

**Figure 3:**
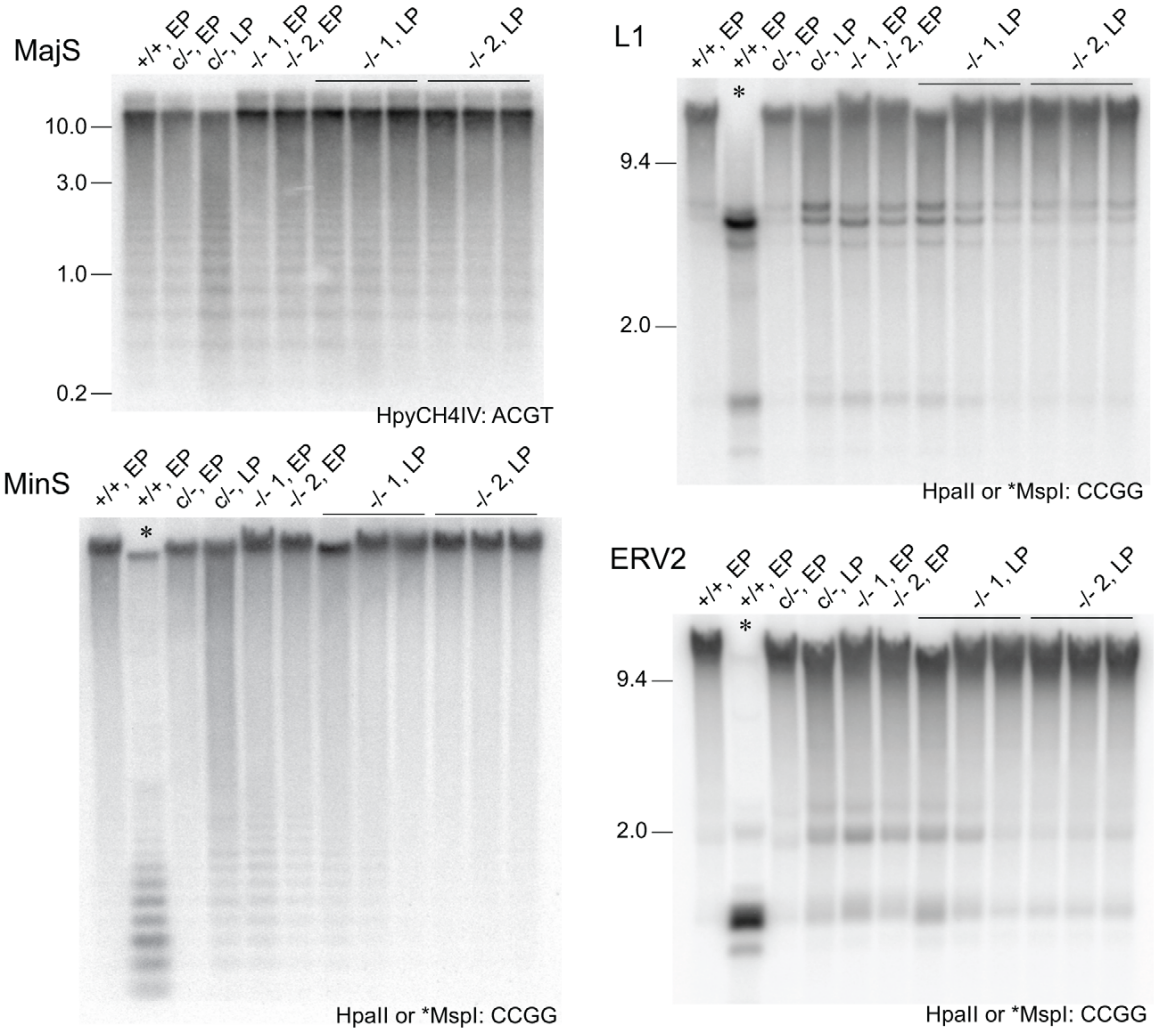
Stability of DNA methylation in DICER1-deficient ES cells. Methylation-sensitive Southern blots. Details are provided in the legend to Figure 1, except that later passage (LP) cells were also used. Passage (p) numbers were: +/+, EP (p8), c/−, EP (p7), c/−, L (p24), −/− 1, EP and −/− 2, EP (p5), −/− 1, LP and −/− 2, LP (p20). Generally, a single passage for +/+ and c/− lines lasted 3 days, while for −/− cells a single passage lasted 5 days. The restriction enzyme and its recognition sequence are at the bottom right of each plate. At CCGG sites, HpaII cuts only when the CpG dyad is unmethylated. The isoschizomer MspI can cut when the CpG dyad is fully, hemi- or unmethylated.

Given the dynamics of DNA methylation as summarized above, critically reduced DNMT activity in DICER1-deficient ES cells could result in variable DNA methylation content depending on the length of time the cells have spent in culture. It was not clear if the reduction in de novo *Dnmt* mRNA and DNMT protein in our *Dicer1* ^−/−^ EP lines was sufficient to lead to reduced DNA methylation. Methylation-sensitive Southern blot analysis was therefore used to analyse the degree of methylation at MajS and MinS repeats, and two dispersed repetitive transposable elements (TEs)—long interspersed nuclear element type 1 (L1) and endogenous retrovirus type 2 (ERV2) or intracisternal A particle. No reduction in methylation was seen at the MajS repeats (Figure 3). By contrast, some reduction in DNA methylation was evident at the MinS repeats and L1 and ERV2 elements in the two *Dicer1* ^−/−^ EP lines (Figure 3).

**Figure 4:**
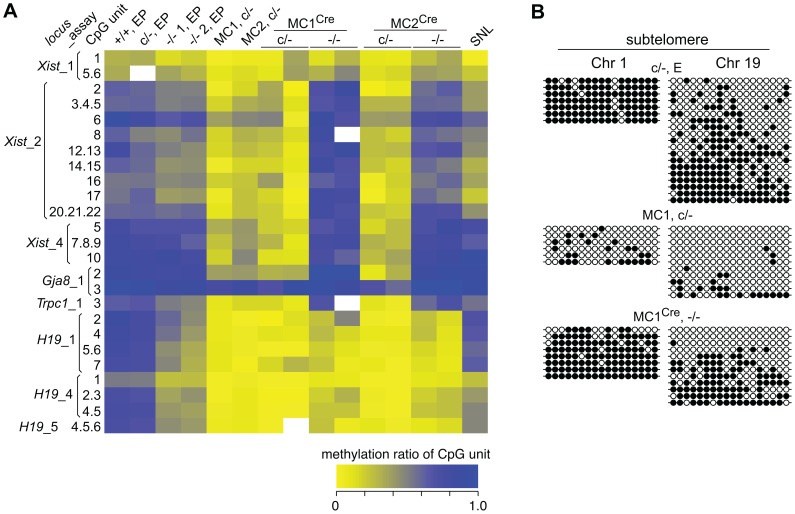
DNA methylation analysis of single copy sequences. (A) Heat-map showing the degree of methylation at CpG units as assayed by EpiTYPER. For example, a yellow rectangle indicates a small fraction of the CpG unit was methylated. Actual values obtained are provided (Dataset S1). The *Xist*_1 and _2 assays correspond to CpG-rich region 2 within exon 1, while the *Xis*t_4 assay corresponds to CpG-rich region 1 directly adjacent to the 5′ end of exon 1. These regions correspond to the *Xist* promoter [16]. The *Gja8* and *Trpc1* assays correspond to 5′ upstream regions of these genes. The *H19*_1 assay corresponds to the *H19* promoter region, while the *H19*_4 and _5 assays correspond to the insulin-like growth factor 2 (*Igf2*)/*H19* imprinting control region located 5′ upstream of *H19*. In EpiTYPER assays, CpGs are analysed as ‘units’, that is, the methylated fraction measured may correspond to one CpG, e.g. (*Xist*_2, 8), or be the average of more than one CpG, for example (*Xist*_2, 20.21.22). SNL, mitomycin C-inactivated SNL feeder cells. (B) Bisulphite sequencing assays. Each row represents a sequenced clone. Closed and open circles represent a methylated and non-methylated CpG, respectively.

Selected single copy sequences in *Dicer1* ^−/−^ EP lines were also analysed for DNA methylation using the EpiTYPER assay (Sequenom, San Diego, CA, USA). This assay is similar to bisulphite sequencing in that DNA is converted using sodium bisulphite and then amplified [28], but differs in that the PCR product is subjected to cleavage and analysis by mass spectroscopy [29]. Sequences assayed were the *Xist* promoter region, reported to show reduced DNA methylation in XY *Dicer1* ^−/−^ ES cells [16], two regions reported to be heavily methylated in WT ES cells—the upstream regions of the *Gja8* (gap junction protein, alpha 8) and *Trpc1* (transient receptor potential cation channel, subfamily C, member 1) genes [30], and the promoter and imprinting control region of the imprinted *H19* (H19 fetal liver RNA) gene. The latter imprinted sequences were included as a reference: differential DNA methylation and monoallelic expression at imprinted regions can be lost over passage in ES cells and cannot be regained, this being shown for the *H19* promoter [31], [32]. This is presumably because the imprintable CpGs are permissive for de novo methylation only during germ cell development, and therefore gradually lose methylation in ES cells as would any other sequence if it were not a target for de novo methylation. In the *Dicer1* ^−/−^ EP lines, some reduction in DNA methylation was evident at some CpG units in the *Xist*_2 region, while no reduction was seen for the *Xist*_1 and _4 regions (Figure 4A). In a previous study, substantial reductions were seen at all of these *Xist* regions using the EpiTYPER assay [16]. A reduction was also seen for the *Trpc*_1 CpG unit, and more noticeable demethylation at all of the CpG units in the *H19*_1, _4 and _5 regions (Figure 4A). For the *H19* CpG units, the loss of methylation is expected as the EP clonally-derived *Dicer1* ^−/−^ cell lines are effectively at much higher passage than the *Dicer1* ^c/−^ EP parental cell line.

To determine if the reduction in DNA methylation seen in the two *Dicer1* ^−/−^ EP lines was the beginning of a generalized reduction of DNA methylation with passage—as would be expected if DNMT activity were compromised—they were propagated for a further 60 days involving at least 15 additional passages. All cell lines reached passage 20 or above, which is sufficient to result in substantial loss of global DNA methylation in the absence of de novo DNMT activity [27]. By passage 15, each of the two cultures of *Dicer1* ^−/−^ lines had developed a strong tendency for differentiation, but this was eliminated by picking and expanding undifferentiated clones. The parental *Dicer1* ^c/−^ line was passaged alongside the two *Dicer1* ^−/−^ lines and was always phenotypically normal. After this passaging regime, or at later passage (LP), DNA was harvested from three sublines for each of the two *Dicer1* ^−/−^ lines, and from the *Dicer1* ^c/−^ line. In the *Dicer1* ^−/−^ LP sublines, the DNA methylation levels had remained stable for the MajS repeats, or even increased slightly for the MinS repeats and the L1s and ERV2s (Figure 3). In the parental *Dicer1* ^c/−^LP line, a loss of methylation at the MinS and two dispersed repeats occurred (Figure 3). This effect seen in the *Dicer1* ^c/−^ line—which would be expected to behave similarly to WT, may reflect a normal level of fluctuation. While, the degree of stability of DNA methylation in WT cells has not been studied extensively, we note that WT cell lines used in other studies exhibit small reductions of methylation at repetitive sequences [9], [13], [14]. The absence of any progressive DNA methylation loss at repetitive elements in *Dicer1* ^−/−^ ES cells with passage indicated that longer-term DNMT activity was essentially intact in these si- and miRNA-deficient ES cells. However, the reductions in DNA methylation seen at the MinS repeats and TEs in *Dicer1* ^−/−^ EP lines and in the parental *Dicer1* ^c/−^ LP line (Figure 3 and Figure 4A) did correlate with a low level of DNMTB (Figure 2). To further examine this phenomenon, we devised a rebound DNA methylation assay to test the ability of EP *Dicer1* ^−/−^ ES cells to methylate their DNA.

### The rebound DNA methylation assay

**Figure 5:**
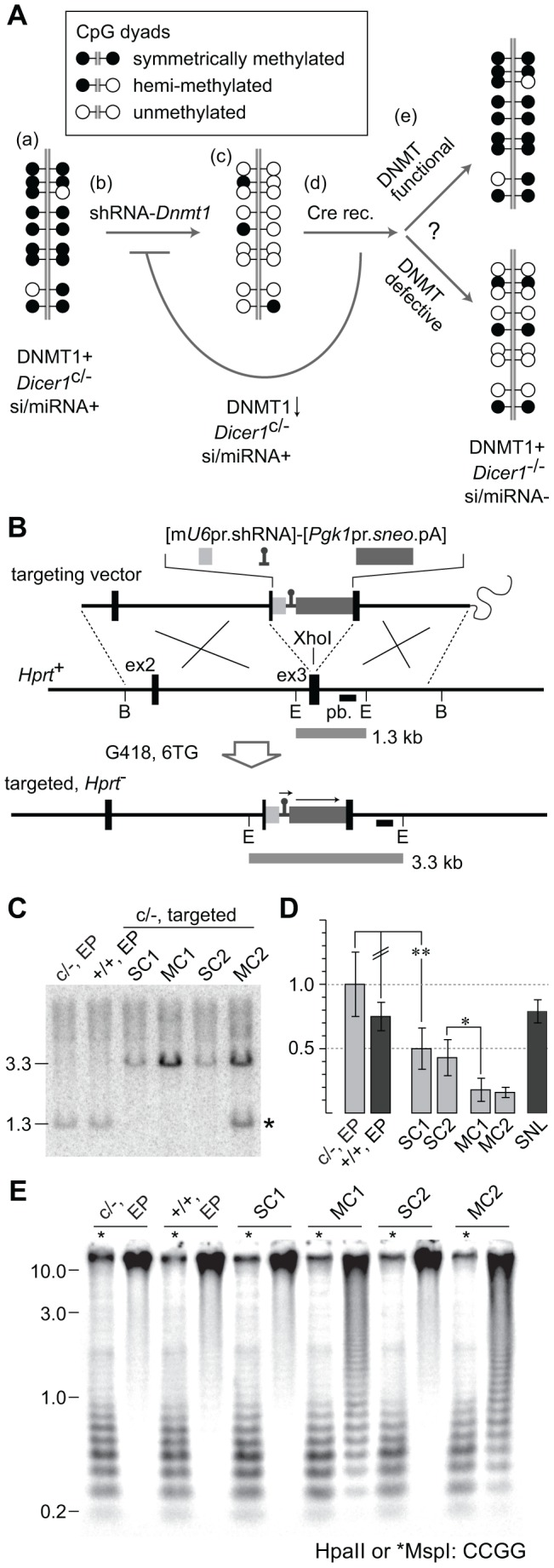
Experimental strategy to test rebound DNA methylation after *Dicer1* ablation. (A) (a) The parental *Dicer1* ^c/−^ XY ES cell line is normally methylated. (b) A construct containing an shRNA targeting *Dnmt1* mRNA (shRNA-*Dnmt1*) is homologously recombined into the X-linked *Hprt* locus. (c) The integrated shRNA effects substantial reduction in genome-wide DNA methylation content, although a background of DNA methylation is retained due to ongoing de novo DNMT activity. (d) Recombinant clones are transiently transfected with Cre to ablate the *Dicer1* ^c^ allele. After ablation, clearing of residual DICER1 and si- and miRNAs, as well as restoration of functional DNMT1 activity, is expected to occur within a few cell divisions. (e) DNA methylation would potentially be re-established over ∼5 passages, corresponding to ∼25 cell divisions through the combined action of the DNMT system as a whole. The extent of rebound DNA methylation serves as an indicator of the dependency of this process on DICER1 activity and the presence of si- and miRNAs. (B) The targeting vector consisted of a 6.8 kb BamHI (B) genomic fragment of *Hprt* spanning exons 2 and 3 (ex2 and ex3). The m*U6* promoter (pr) driving the shRNA and a *sneo* selection cassette were inserted into ex3. The sequence of the shRNA cassette is provided (Text S1). All clones surviving G418 and 6TG dual selection are expected to be targeted. pb, probe used in Southern blot, hybridizing to EcoRI (E) bands as indicated by horizontal bars. (C) Southern blot to detect gene targeting. WT bands are seen in the parental *Dicer1* ^c/−^ line, and in a *Dicer1* ^+/+^ line. An increase in band size is occurs when the single *Hprt* sequence is replaced by a single copy (SC) or multiple copy concatemer (MC) of the targeting vector. In addition to the mutant band, clone MC2 clone shows a WT band of normal intensity (*). This results from recombination occurring in the 5′ arm, rather than in the 3′ arm, of the terminal vector in the concatemer. (D) qPCR of *Dnmt1* mRNA. All values were calibrated to that obtained for the *Dicer1* ^c/−^ line, adjusted to 1.0. SNL, mitomycin C-inactivated feeder cells. Other details as above. (E) Southern blot to detect methylation levels at MinS repeats. Details as above.

The rebound DNA methylation assay is depicted (Figure 5A). Genomic DNA in *Dicer1* ^c/−^ (c, Cre/*loxP* conditional null allele; -, null allele) ES cells is largely stripped of methylation using a shRNA complementary to *Dnmt1* mRNA. These ES cells are then exposed to Cre recombinase, converting them to *Dicer1* ^−/−^ while simultaneously abrogating shRNA processing and restoring *Dnmt1* mRNA. Then, the ability of these cells to regain DNA methylation provides a readout of functional DNMT activity. This activity is the combined functional activity of DNMTs 1, 3A, 3B and 3L, in a si- and miRNA-deficient environment. Rebound DNA methylation could not occur unless all components of this DNMT system are essentially intact [21], [24].

To strip DNA methylation, a m*U6*-shRNA complementary to *Dnmt1* mRNA was targeted to the X-linked housekeeper *Hprt* (hypoxanthine guanine phosphoribosyl transferase) gene via sequential G418 and 6-thioguanine (6TG) selection (Figure 5B). This locus is expression-permissive for targeted cassettes, including shRNA cassettes [33], [34]. Following electroporation, ∼20 dual drug resistant clones derived from the parental *Dicer1* ^c/−^ line were obtained per 10 cm plate. This was a ∼10-fold higher frequency of clone recovery compared to our previous experience using the same homology arms for *Hprt* [33], and could be related to the opposite transcriptional orientation of the positive selection cassette, or use of the *sneo* rather than the *neo* selection cassette. After two passages—with growth of the initial clone being the first passage, half of the cells were frozen while the other half were used for DNA extraction. Of 16 clones analysed by Southern blot, band intensity relative to DNA loading indicated that 12 clones were single copy (SC) recombinants, while four clones were multiple copy (MC) recombinants. The latter derive from concatemeric insertion of the complete targeting vector including vector sequences, this being expected when no negative selection cassette is present. Two SC and two MC clones were selected for further study. Relative band intensity indicates the number of vector copies integrated in MC clones was ∼four (Figure 5C).

Frozen cells were thawed and grown for another 2 passages, corresponding to 5 passages after DNA integration, then total RNA and genomic DNA extracted for qPCR and DNA methylation analysis. qPCR revealed that the level of reduction in *Dnmt1* mRNA content correlated with SC or MC integration. Relative to the parental cell line, the two MC lines showed more than 80% reduction in mRNA while the former showed ∼50% reduction (Figure 5D). Immunoblots showed that DNMT1 was low in the two MC lines relative to the *Dicer1* ^+/+^ EP line (Figure 2). Also noticeable in these MC lines was a considerable reduction in the amount of *Dnmt3b* mRNA relative to the parental *Dicer1* ^c/−^ EP line (Figure 1A). While this could have resulted from off-targeting activity of the introduced shRNA, this would not present a problem for our strategy as we were trying to achieve as much reduction of DNA methylation as possible before ablation of *Dicer1* activity.

### Reduction of genomic DNA methylation content in MC sublines

**Figure 6:**
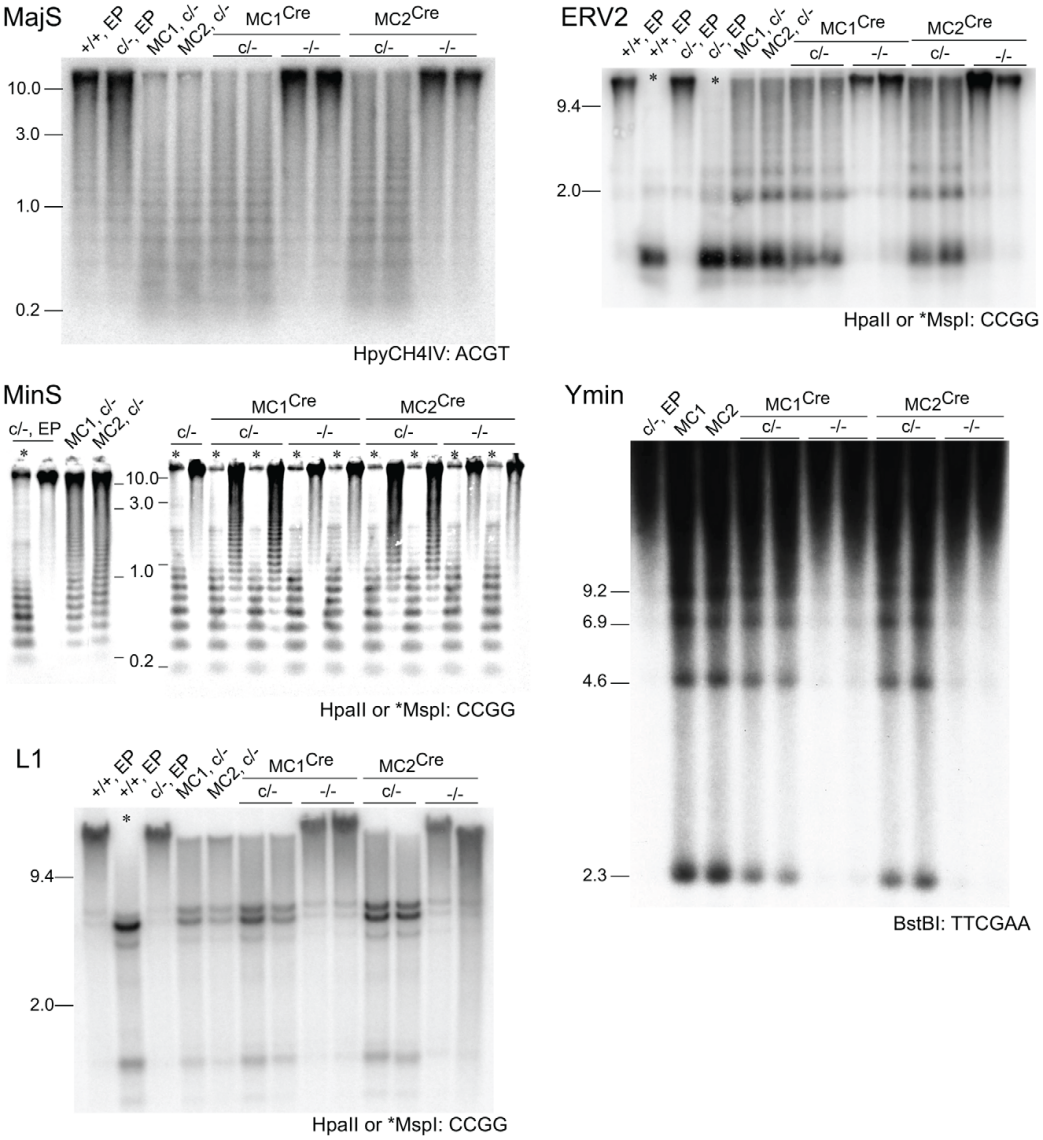
Rebound DNA methylation at repetitive sequences after *Dicer1* ablation. Southern blots. MC1^Cre^ and MC2^Cre^ are sublines derived from Cre transfection of line MC1 and MC2 respectively. Other details are as provided in the legends to previous figures.

Genomic DNA isolated from the SC and MC lines was assayed for DNA methylation at MinS repeats. No reduction in methylation was seen in the two SC lines (Figure 5E), and these were not analysed further. By contrast, substantial loss of DNA methylation in the two MC lines was seen. Other sequences assayed in these MC lines were (i) localized repeats —MajS and Y Chr centric repeats (Ymin), using methylation-sensitive Southern blots, (ii) dispersed repeats—L1s and ERV2s, also using Southern blots, and (iii) selected single copy sequences—as described above, using bisulphite sequencing and EpiTYPER. All repeat and single copy sequences displayed a substantial loss of DNA methylation (Figure 4A, 4B and Figure 6), with the exception of CpG unit 3 within the *Gja8*_1 region (Figure 4A). This retention indicates that there is an unusually high frequency of maintenance methylation effected by DNMT3A or DNMT3B at this site. This atypical CpG unit was only 121 bp from another CpG unit, *Gja8*_1 unit 2, that displayed the typical DNA methylation dynamics associated with loss of DNMT1. In general, the degree of DNA methylation loss was similar to that seen in *Dnmt1* ^−/−^ ES cells and in ES cells in which the same shRNA sequence was introduced by lentivirus [35].

### Effect of *Dicer1* ablation on *Dnmt* mRNA and DNMT protein content

The two targeted *Dicer1* ^c/−^ lines MC1 and MC2 were transiently transfected with a Cre expression vector then plated at low density to obtain clonal growth. For each of the MC1 and MC2 transfections, normally growing (presumptive *Dicer1* ^c/−^) and slow growing (presumptive *Dicer1* ^−/−^) sublines were picked, propagated until passage 5, then total RNA and genomic DNA extracted and genotype confirmed by Southern blot. Three each of control MC^Cre^ *Dicer1* ^c/−^ and experimental MC^Cre^ *Dicer1* ^−/−^ sublines were selected for qPCR. As expected, the amount of *Dnmt1* mRNA remained low in the former and was restored in the latter to the level seen in the parental *Dicer1* ^c/−^ cell line. This restoration was to a level higher than that seen in the two *Dicer1* ^−/−^ EP lines (Figure 1A). In the three MC^Cre^ *Dicer1* ^−/−^ sublines the amount of *Dnmt3a* mRNA was higher than in the *Dicer1* ^−/−^ EP lines, although was not significantly different to that in the parental *Dicer1* ^c/−^ cell line. Similarly, the amount of *Dnmt3b* mRNA in the three MC^Cre^ *Dicer1* ^−/−^ sublines was higher than in the *Dicer1* ^−/−^ EP lines. However, this remained lower than in the parental *Dicer1* ^c/−^ cell line (Figure 1A). *Dnmt3l* and *Rbl2* mRNA content was no different in MC^Cre^ *Dicer1* ^−/−^ sublines compared to the *Dicer1* ^−/−^ EP lines, being elevated ∼twofold and reduced ∼fivefold relative to the parental *Dicer1* ^c/−^ EP line, respectively (Figure 1A).

Immunoblots (Figure 2) confirmed that there was a marked rebound in DNMT1 level in the MC^Cre^ *Dicer1* ^−/−^ sublines, although this did not reach the level seen in the *Dicer1* ^+/+^ EP line. As expected, the amount of DNMT1 remained low in the MC^Cre^ *Dicer1* ^c/−^ sublines (right blot). DNMT3A2 levels were lower in the MC^Cre^ *Dicer1* ^−/−^ sublines compared to the MC^Cre^ *Dicer1* ^c/−^ sublines and the parental *Dicer1* ^c/−^ EP line (right blot). DNMT3B levels in MC^Cre^ *Dicer1* ^−/−^ sublines were ∼80% of that seen in the parental *Dicer1* ^c/−^ EP line (right blot). Also, there was a marked increase in the amount of DNMT3B in the *Dicer1* ^−/−^ LP sublines compared to the *Dicer1* ^−/−^ EP cells from which they were derived (left blot). Thus, DNMT3B levels had increased over passage, but only to ∼50% the level in the *Dicer1* ^+/+^ EP line. DNMT3L and RBL2 levels in the MC^Cre^ *Dicer1* ^−/−^ sublines were ∼70% and ∼80% that detected in the *Dicer1* ^+/+^ EP line, respectively (right blot). Also, RBL2 was clearly lower in these MC^Cre^ *Dicer1* ^−/−^ sublines compared to the MC^Cre^ *Dicer1* ^c/−^ sublines (right blot). Thus, again, DNMT3L and RBL2 protein levels did not reflect the amounts of mRNA present.

### Effect of *Dicer1* ablation on rebound DNA methylation

In our experimental system, at the point of Cre-mediated conversion from *Dicer1* ^c/−^ to *Dicer1* ^−/−^, ES cells were globally hypomethylated. From this stage, only a few cell divisions (one passage), would be required for depletion of si- and miRNAs [36]. This would include depletion of the introduced siRNA that targets *Dnmt1* mRNA, therefore there would be a lag in the restoration of DNMT1 protein. Given these kinetics, and that complete restoration of global DNA methylation content requires 5 passages with full DNMT activity [37], a complete rebound in DNA methylation in the experimental *Dicer1* ^−/−^ sublines must be realized largely in the absence of si- and miRNAs. In the four experimental *Dicer1* ^−/−^ sublines, two each derived from lines MC1 and MC2, after a total of 5 passages, essentially full restoration of DNA methylation content occurred at all sequences examined—MajS, MinS, Ymin, L1 and ERV2 repeats (Figure 6). In addition, using immunofluorescence, the MajS-specific mark H3K9me3 and downstream heterochromatic marks CBX5 (chromobox homolog 5 (Drosophila HP1α)) and H4K20me3 appeared to be as prevalent in one *Dicer1* ^−/−^ subline examined as in controls (Figure S1A–S1C). Indeed, the labelling of these marks appeared to be highest in the *Dicer1* ^−/−^ cells and could be indicative of some alteration of chromatin structure in the form of a greater concentration or accessibility of marks. This could be related to the increased transcription seen at these sequences [9], [13]. Essentially complete rebound in DNA methylation was also seen for single copy sequences that had lost methylation due to DNMT1 deficiency, these being the three *Xist* regions, the *Trpc1*_1 region, and the two subtelomeric regions. The exceptions were the three *H19* regions (Figure 4A and 4B), no rebound being expected given that the methylation of these sites is germ line-specific. Overall, the results indicate that functional DNMT activity is normal in *Dicer1* ^−/−^ EP ES cells.

## Discussion

DICER1-deficient mouse ES cells have often been found to be deficient in DNA methylation. These observations have been predominantly of localized and dispersed repeats which constitute the large majority of methylated CpG dyads in the mammalian genome [4]. While a loss in RNAi-mediated pathways of heterochromatin formation could be involved, particularly at MajS repeats, the predominant cause has been proposed to be a downregulation in *Dnmt* gene activity primarily due to the loss of *Mir290* cluster miRNAs.

In re-examining these possibilities, we assayed functional DNMT activity in *Dicer1* ^−/−^ ES cells using two experimental strategies. The first strategy was the continued passage of *Dicer1* ^−/−^ cells in order to assay for the longer-term stability of functional DNMT activity. *Dicer1* ^−/−^ EP lines had reduced DNA methylation at the MinS repeats and TEs. The significance of this effect in respect to DICER-deficiency is unclear, as similar reductions were also seen in the parental *Dicer1* ^c/−^ LP cell line, and is sometimes evident in WT cell lines in other studies. The reductions were correlated with low amounts of DNMT3B, and this may have been causal. However, in one of these cell lines the DNMT3A2 level was not reduced and this enzyme alone is sufficient for normal methylation at dispersed repeats [21]. In any event, if the methylation reductions in the *Dicer1* ^−/−^ EP lines were due to an irreversible and critical reduction in DNMT activity, then the levels of methylation would further decrease. Instead, under this regime, no line lost methylation at TE or MajS sequences on passage. Thus, over prolonged passage, almost certainly attaining higher passage numbers in *Dicer1* ^−/−^ ES cells than have previously been investigated, fully functional DNMT activity was present.

The results obtained with LP cells did not clearly indicate if, specific to EP, there was some compromise in DNMT activity. There would seem no practical method by which to directly measure this activity in these cell lines per se. Therefore, a second experimental strategy was devised to determine the amount of functional DNMT activity that is generally present in *Dicer1* ^−/−^ ES cells very soon after their origination. After stripping the genome of DNA methylation, then ablating *Dicer1* function, we found that all methylation-permissive sequences were remethylated at normal kinetics to full capacity, these being satellites, TEs, and non-imprinted single copy sequences. These results show that full DNMT activity is generally present in *Dicer1* ^−/−^ ES cells in the earliest passages after their origination.

Our examination of *Dnmt* and *Rbl2* mRNA levels and their respective protein levels in *Dicer1* ^−/−^ ES cells are not in support of previous studies which concluded that increased amounts of the transcriptional repressor RBL2 leads to downregulation of *Dnmt* gene activity [12], [15]. These conclusions were based predominantly on the finding that *Rbl2* mRNA was increased, and on the presumption that RBL2 protein was also increased. While we also saw increased *Rbl2* mRNA in our *Dicer1* ^−/−^ lines, immunoblots revealed that RBL2 protein was actually relatively low. Thus, the cause of *Dnmt* transcriptional downregulation in *Dicer1* ^−/−^ ES cells would appear to lie elsewhere. Reduced levels of mRNA for all three *Dnmts* involved in de novo DNA methylation were found in our *Dicer1* ^−/−^ EP lines. At the protein level, only DNMT3B was reduced, which was inconsistent with other studies that saw only DNMT3A to be reduced in *Dicer1* ^−/−^ ES cells [15], [16]. Despite these reductions, it should be noted that ES cells are able to retain high levels of DNA methylation at least at TEs despite a substantial reduction in total de novo DNMT activity: *Dnmt3a* ^−/−^ or *Dnmt3b* ^−/−^ ES cells are not generally DNA methylation deficient. Each is able to de novo methylate introduced retroviral sequences at normal rates, and each is stably methylated at TEs. However, they can be deficient at particular sites according to site preference. For example, DNMT3A preferentially methylates MajS repeats and *Xist* promoter sequences, while DNMT3B preferentially methylates MinS repeats [21], [38].

Our results suggest that H3K9me3, that at least in part directs DNA methylation to MajS repeats, remains intact and functional in ES cells in the absence of si- and miRNAs. While these results indicate that RNAi is not indispensable for the establishment and propagation of heterochromatin in ES cells, it remains possible that it plays some role. Relative to fission yeast, eukaryotes probably possess a higher degree of redundancy in pathways that lead to heterochromatin formation. Even in the former, histone deacetylase activity is able to establish constitutive heterochromatin independent of RNAi [39]. At early stages of mouse preimplantation development, heterochromatinization of pericentric repeats is dependent on their transcription and it has not been discounted that siRNAs contribute to the overall mechanism [40]. Indeed, deep sequencing in mouse ES cells has revealed the presence of satellite-specific small ncRNAs, although these are not DICER1-dependent [14]. Further, relative to somatic cells, which can show substantial defects in heterochromatin on loss of DICER [41], the mammalian POU5F1/OCT4-positive lineage may rely less on RNAi for heterochromatin establishment and propagation.

Previously, it was shown that the transfection of *Dicer1* ^−/−^ ES cells with *Dnmt* expression cassettes restored a deficiency in genomic DNA methylation [12]. While this result is consistent with there being a critical reduction in DNMT activity in *Dicer1* ^−/−^ cells, the transfected *Dnmt* cDNAs were substantially over-expressed relative to WT, therefore excess DNMT activity could have overridden other mechanisms acting to reduce DNA methylation. There is considerable evidence that the over-expression of DNMTs results in hypermethylation with oncogenic consequences, for example [42]–[44]. Further, production of stable transfectants requires considerable further propagation of the cells, and as demonstrated here, this alone can result in increased DNA methylation in *Dicer1* ^−/−^ cells.

Defective DNA methylation is also a feature of XX ES cells [27], [45], thereby bearing a resemblance to the phenomenon being examined here. However, the effect in XX ES cells can be substantial, with up to 50% reduction in the total amount of DNA methylation relative to XY and XO ES cells. This reduction does appear attributable to lowered levels of DNMT3A and DNMT3B, in particular the former, as the restoration of physiological amounts of these proteins through stable transfection resulted in normalization of DNA methylation status [45]. The mechanism causing reduced *Dnmt3a* and *Dnmt3b* expression in XX ES cells is unknown, although it was speculated that the loss may be attributable to the excessive expression of an X-linked de novo *Dnmt* repressor [45]. This repressor could not be the *Mir290* gene family, as this maps to Chr 7. Overall, the demethylation phenomenon in XX ES cells is more pronounced than that seen in *Dicer1* ^−/−^ ES cells, and appears to involve different mechanisms.

We have obtained no convincing evidence that the reductions in DNA methylation sometimes seen in *Dicer1^−/−^* ES cells, and also seen here, are the result of a critical reduction in DNMT activity. Certainly, we show that reduced DNMT activity, and a reduced capacity for DNA methylation, are not consistent features of DICER1-deficient ES cell lines. It is conceivable that there could be other mechanisms by which DNA methylation could be lost in ES cells lacking DICER1. These ES cells are defective in the G1-S phase transition due to the loss of *Mir290* family miRNAs, and consequently have a greatly extended cell cycle [46]. This extended time in phase G1 might result in a higher probability of stochastic changes in DNA methylation patterns that could then be propagated—particularly if they offered a growth or survival advantage in culture. Also, the loss of miRNAs might result in a dysregulation of gene expression that works against the stable retention of DNA methylation. For example, this could involve DNMT mislocalization, or even an enhancement of DNA demethylation pathways. While the latter are not clearly defined, there is strong evidence for their existence in mouse ova, primordial germ cells and some somatic cells [47]. Irrespective of the actual mechanism for the loss of DNA methylation seen in DICER1-deficient ES cells, the effect appears to be sporadic and transient, and is not a salient or major epigenetic alteration resulting from loss of the RNAi machinery.

## Materials and Methods

### Ethics statement

This work was performed in accordance with the ‘Australian Code of Practice for the Care and Use of Animals for Scientific Purposes’ published by the National Health and Medical Research Council (Australia) and as approved by the Animal Ethics Committee of The University of Melbourne.

### Derivation of ES cells, gene targeting, and ablation of *Dicer1*

ES cell lines were derived from (*Dicer1* ^c/c^ ♀×*Dicer1* ^+/−^ ♂) matings in mouse strain 129S1/SvImJ and propagated using mitomycin C inactivated STO-*neo*-LIF (SNL) feeder cells [18], [48]. One euploid XY *Dicer1* ^c/−^ ES cell line—named DC8—was selected for further experiments. *Dicer1* ^c/−^ ES cells were mutated to *Dicer1* ^−/−^ by electroporating 10^7^ cells with 25 µg of the circular pCAGGS-Cre vector [49]. One thousand cells were seeded onto 6 cm plates and after 8 days of growth colonies were picked into wells and expanded. In Southern blots, *Dicer1* ^+/+^, *Dicer1* ^c/−^ and *Dicer1* ^−/−^ genotypes were identified after EcoRI digestion by a 7.7 kb, a 4.0 and 5.6 kb, and a 5.6 kb band, respectively, using a 0.75 kb probe amplified from genomic DNA using primers 5′-CCAA GAAC CCAT AGCT TCCC ATC-3′ and 5′-TCAG ACAA CTGT TACG GTGT CGTG-3′. The *Dicer1* ^+/+^ cell line used was 2A: euploid, XY, strain 129S1/SvImJ [50].

The *Hprt* targeting construct consisted of a 6.8 kb genomic fragment in which an expression cassette was cloned into a unique XhoI site in exon 3 [33], [51]. The m*U6* promoter sequence identical to that in the pSilencer 1.0 vector, (Ambion), together with cloning sites, was synthesized (Integrated DNA Technologies, Coralville, IA, USA). The short hairpin sequence [35] was cloned into inverted BsmBI sites, and a G418 resistance cassette—mouse *Pgk1* (phosphoglycerate kinase 1) promoter driving a synthetic *neo* coding sequence (*sneo*) [52] and the bovine growth hormone poly(A) signal sequence—was inserted downstream into a unique AscI site. These cassettes were inserted in the same transcriptional orientation as *Hprt* (Figure 4A). Ten million DC8 ES cells electroporated with 25 µg of the linearized targeting vector (Figure 3A) were plated per 10 cm plate containing SNL feeder cells that are G418 and 6TG resistant. G418 selection (175 µg/mL active weight) began 1 day after plating, while 6TG selection (10 µg/mL) was initiated after another 4 days with G418 selection being continued. Dual resistant clones were picked after another 3 days.

### Isolation of genomic DNA and RNA

At the final passage before harvesting, all cells in a confluent 6 cm plate of ES cells were passaged into one gelatinized 10 cm plate without feeder cells and grown for 2 days. ES cells were then further enriched by differential adherence, resulting in <2% level of SNL feeder cell contamination [31]. For genomic DNA extraction, these cells were pelleted, resuspended in 4 mL of phosphate buffered saline, then four pellets of equal size derived in four 1.5 mL tubes. Each pellet was incubated in 0.5 mL of lysis buffer (50 mM Tris-HCl pH 8.0, 100 mM NaCl, 100 mM EDTA, 1% SDS; with no protease added) (37°C, 2 h or room temperature, 16 h or longer). To pellet protein and SDS, 0.17 mL of 10 M ammonium acetate was added, the tube vortexed then centrifuged (14,000× *g*,10 min). The supernatant was removed and mixed thoroughly with an equal volume of isopropanol to precipitate DNA, then a centrifuge pulse applied. The pellets were washed in 1 mL 70% ethanol. Air dried pellets were resuspended in 50 µL of TE buffer. RNA was isolated using TriReagent (Ambion).

### qPCR

For assay of miRNA content, TaqMan MicroRNA assays (Applied Biosystems) were used. Ct values were normalized to values for *Rps7* (ribosomal protein S7) obtained using a SYBR Green assay as described [53]. For assay of mRNA content, TaqMan qPCR assays were designed using PrimerExpress software (Applied Biosystems) for use in multiplex reactions: 4-plex (*Rps7*, *Alpl*, *Dnmt1*, *Dnmt3a* mRNAs), 4-plex (*Rps7*, *Alpl*, *Dnmt3b*, *Dnmt3l* mRNAs), and 3-plex (*Rps7*, *Alpl*, *Rbl2* mRNAs). The *Rps7* transcript is ubiquitous while the *Alpl* (alkaline phosphatase, liver/bone/kidney) gene is expressed at a relatively high level in ES cells and germ cells. For each sample, two reverse transcription (RT) reactions with oligo-dT and triplicate PCR reactions for each RT were performed, yielding six Ct values. These values were converted to the relative amount of mRNA by multiplying the fold increase in product per cycle (equivalent to amplification efficiency) to the power of ΔCt. Values were normalized using combined *Rsp7* and *Alpl* values. The TaqMan qPCR primers and probe for *Dnmt3b* mRNA detected only the *Dnmt3b1* and *-2* isoforms, these being the only isoforms encoding active protein, while those for *Dnmt3a* mRNA detected the two existent isoforms, *Dnmt3a1* and *-2*, each encoding an active protein [21]. Primer and probe (Integrated DNA Technologies) sequences are provided (Text S1).

### DNA methylation analysis

For Southern blots, genomic DNA was digested for 6 h with a fivefold excess of restriction enzyme (New England Biolabs, Ipswich, MA, USA) in a minimum volume. This resulted in complete digestion as confirmed in blots hybridized with a probe specific for mitochondrial DNA. Digested DNA was loaded at 1 µg/well for repetitive targets, or 5 µg/well for single copy targets. Blots were performed with Hybond XL membrane according to the supplied protocol (GE Healthcare), except that the pre- and hybridization solution was 5× SSPE, 5× Denhardt's solution, 1% SDS. All gels were depurinated. Blots were washed twice (15 min, 65°C) with 0.2× SSC, 0.5% SDS (single copy targets) or 0.1× SSC, 0.5% SDS (repetitive targets). Probes: Detection of homologous recombination at *Hprt*—0.25 kb PCR product made using primers 5′-TCAC TGAT ACTT CATA TCAC A-3′ and 5′-GCCT AAGA ATTG CTAT TGAA T-3′. MajS and MinS—as previously described [54]. L1, pL1-ORF1—0.92 kb EcoRI fragment representing a conserved portion of open reading frame 1 (ORF1), amplified with primers 5′-AACA CTGC TAAA GAGT TACA AGTC C-3′ and 5′-CCGT CCTT GTAT TGGT TTTT TTCT G-3′. ERV2, pERV2-ORF1—0.52 kb EcoRI fragment representing a conserved portion of ORF1, amplified with primers 5′-TTCA GGAC AAGC TATC AGAA G-3′ and 5′-AATG AATG AGTC TGCG CACT G-3′. Sequences of pL1-ORF1 and pERV2-ORF1 inserts were validated by sequencing. Y Chr centromere, pYmin2.3a [55]. Mitochondrial DNA, pMIT1—0.66 kb EcoRI fragment amplified with primers 5′-GCAC ACAC CGCC CGTC AC-3′ and 5′-GGTT TTTT CCGT TCCA GAAG AGC-3′. All amplified fragments were cloned into vector pGEM-T Easy (Promega) and verified by sequencing.

For bisulphite sequencing analysis, genomic DNA isolated as above was converted with sodium bisulphite using the EZ DNA Methylation Kit (Zymo Research, Irvine, CA, USA). Three separate PCR reactions were performed for each sample with a similar number of clones sequenced from each. Sequencing showed that the conversion rate of Cs to Ts in non-CpGs was complete. Primers were designed with MethPrimer <http://www.urogene.org/methprimer/index1.html>. EpiTYPER assays were performed as previously described [56]. Primers were according to the EpiPanel (Sequenom) or designed with EpiDesigner <www.mysequenom.com>.

### Immunoblots and immunofluorescence

Immunoblots were performed with Hybond P membrane according to the supplied protocols (GE Healthcare). Primary antibodies: anti-DNMT1 (Imgenex, San Diego, CA, USA; IMG-261A), Anti-DNMT3A2 (Millipore; 07-2050), Anti-DNMT3B (Abcam, Cambridge, MA, USA; ab122932), Anti-DNMT3L (Millipore; ABD78), Anti-RBL2/p130 (Abcam; ab89457). Secondary antisera: Goat Anti-mouse IgG, HRP conjugate (Millipore; 12-349) or goat Anti-rabbit IgG, HRP conjugate (Millipore; 12-348). Immunofluorescence was performed as previously described [57], [58]. Primary antibodies: Anti-H3K9me3 (Millipore; 07-523), Anti-CBX5 (Millipore; MAB3584), Anti-H4K20me3 (Abcam; ab9053), and CREST (supplied by the Royal Melbourne Hospital, Australia). Secondary antisera: donkey anti-mouse or anti-rabbit Alexa Fluor 488 -594, or -647 (Life Technologies).

## Supporting Information

Dataset S1 DNA methylation analysis of single copy sequences. Numerical data graphically depicted in Figure 4A.
(XLSX)

Figure S1 Immunofluorescence for markers of pericentric heterochromatin. (A) Panels: First row; metaphase spreads were probed with an antibody specific for H3K9me3. Second row; CREST antibody is specific for the centromere. Third row; merge of the two images directly above, concomitant with detection of DNA with DAPI, reveals the pericentric localization of H3K9me3. (B) As for A, except that CBX5 (synonym HP1α) is shown to be localized to the pericentric region. (C) As for A, except that H4K20me3 is shown to be localized to the pericentric region. *Dicer1* ^c/−^ panels at left are the EP parental cell line.
(PDF)

Table S1 Characteristics of *Dicer1* ^−/−^ ES cell lines. Characteristics surveyed are indicated at left. Green, yellow and blue cell background indicates normal, low and high levels or content, respectively. The letters inside the cells indicate the assay method used: BS, bisulphite sequencing assay; ChIP DB, chromatin immunoprecipitation followed by dot blot; COBRA, comined bisulphite restriction analysis; ET, EpiTYPER assay; IB, immunoblotting; IF, immunofluorescence; MA, microarray; NB, northern blot; nd, not done; PCR, polymerase chain reaction; RTPCR, reverse transcription PCR; SB, Southern blot; qPCR, quantitative PCR.
(XLSX)

Text S1 Primers and sequences. Sequences of primers used in qPCR, bisulphite sequencing assays, EpiTYPER assays and stem-loop cloning are shown. For insertion of the shRNA sequence to be driven by the m*U6* promoter, each of two pairs of oligos as shown were preannealed, phosphorylated, then combined and ligated into the inverted BsmBI sites (red highlight). This strategy avoided cloning with full-length oligos containing a stem-loop. The functional guide strand (green highlight), which forms one strand of the siRNA as produced by DICER1 processing of the shRNA, is exactly complementary to the *Dnmt1* mRNA sequence. The G418 selection cassette was cloned into the unique AscI site in the orientation as indicated (>). To complete the targeting vector, the cassette was excised with XhoI and SalI and cloned into the unique XhoI site in exon 3 of the ∼6 kb *Hprt* genomic fragment. The transcription start site is indicated by the G.
(PDF)
