# Supplementary figures and images for: Normal DNA Methylation Dynamics in DICER1-Deficient Mouse Embryonic Stem Cells
Source: PLoS Genet. 2012 Sep 6;8(9):e1002919. doi: 10.1371/journal.pgen.1002919 (PMC3435250; doi:10.1371/journal.pgen.1002919)

**Figure S1-A**

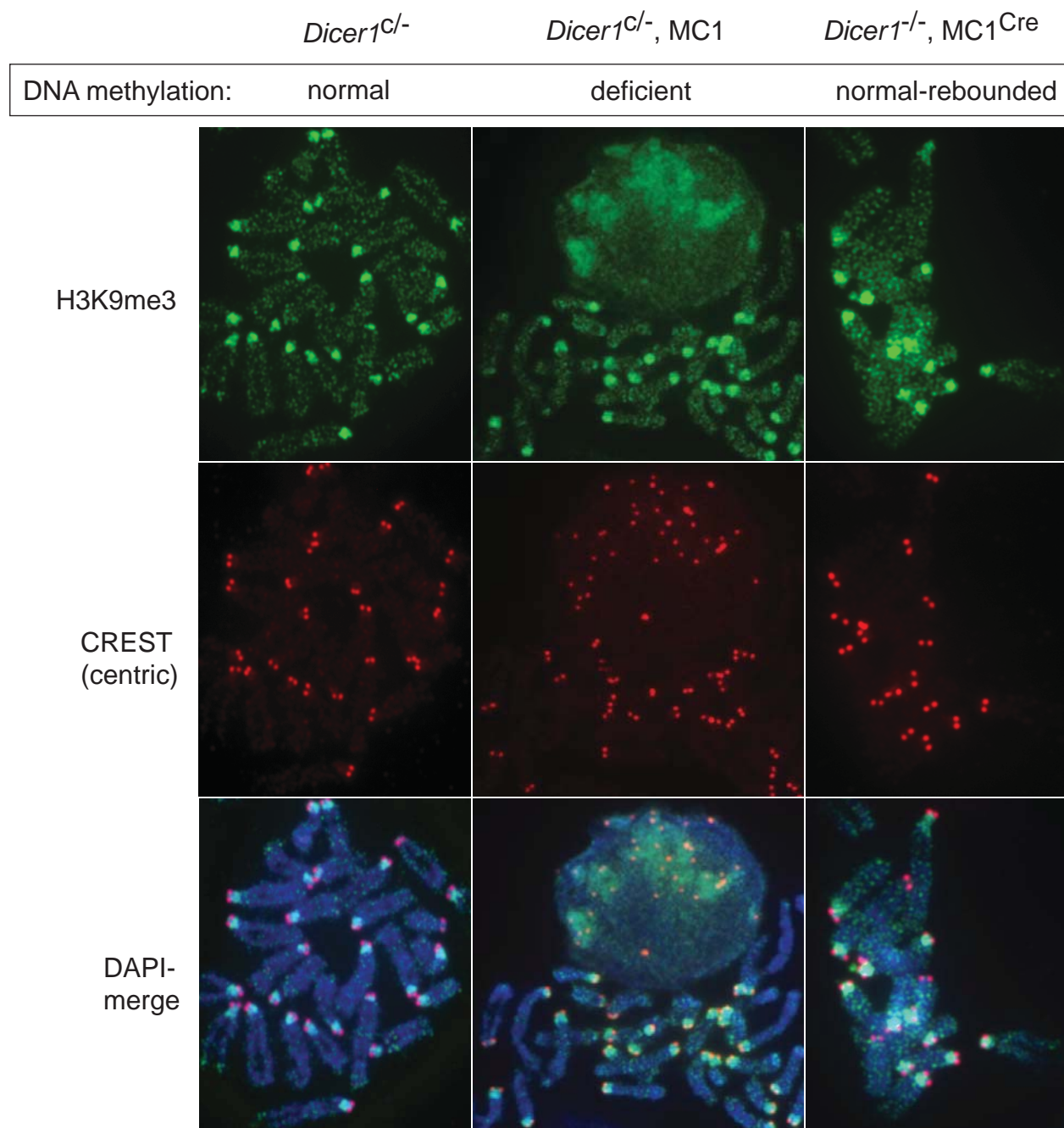

Figure S1-B

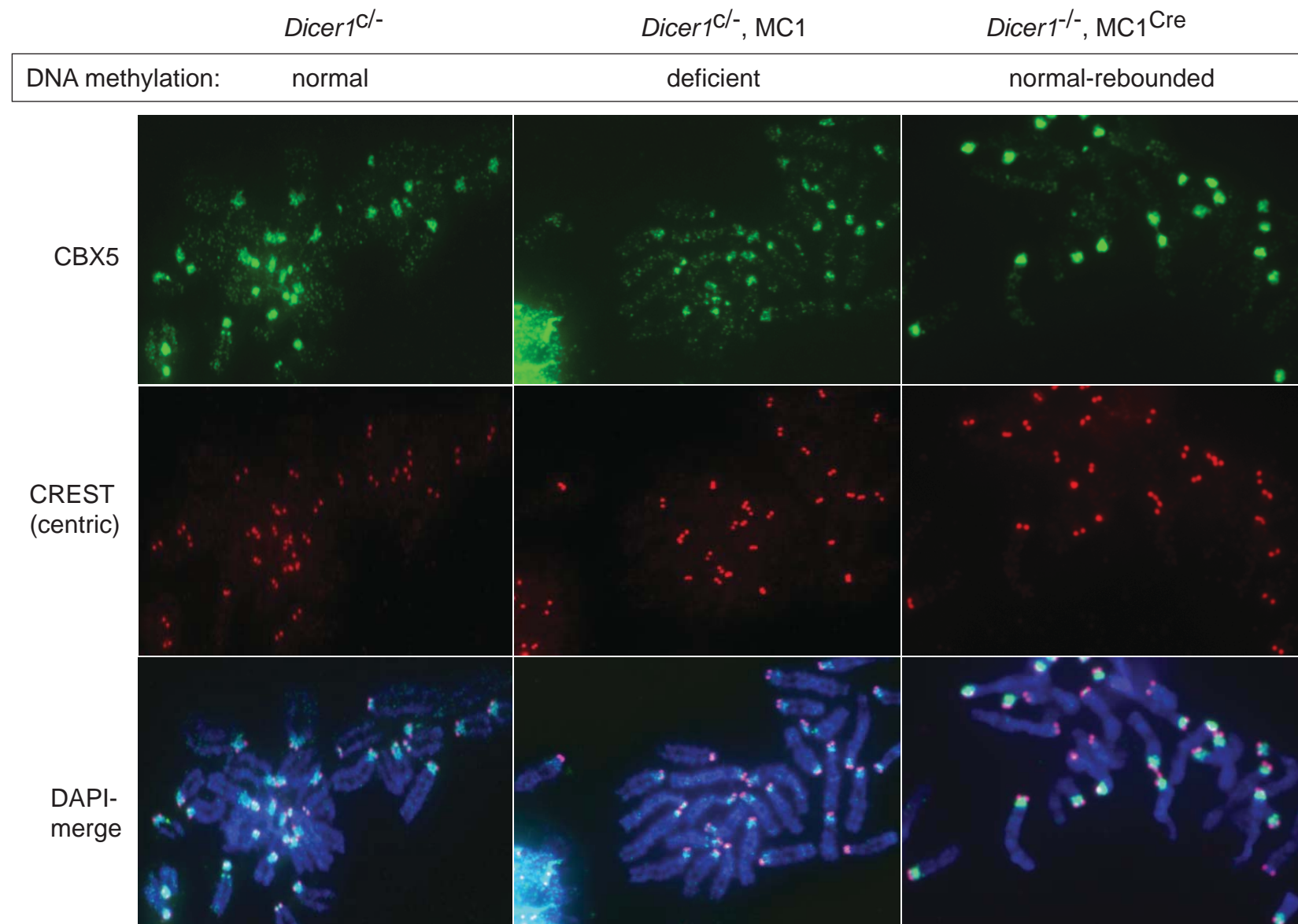

**Figure S1-C**

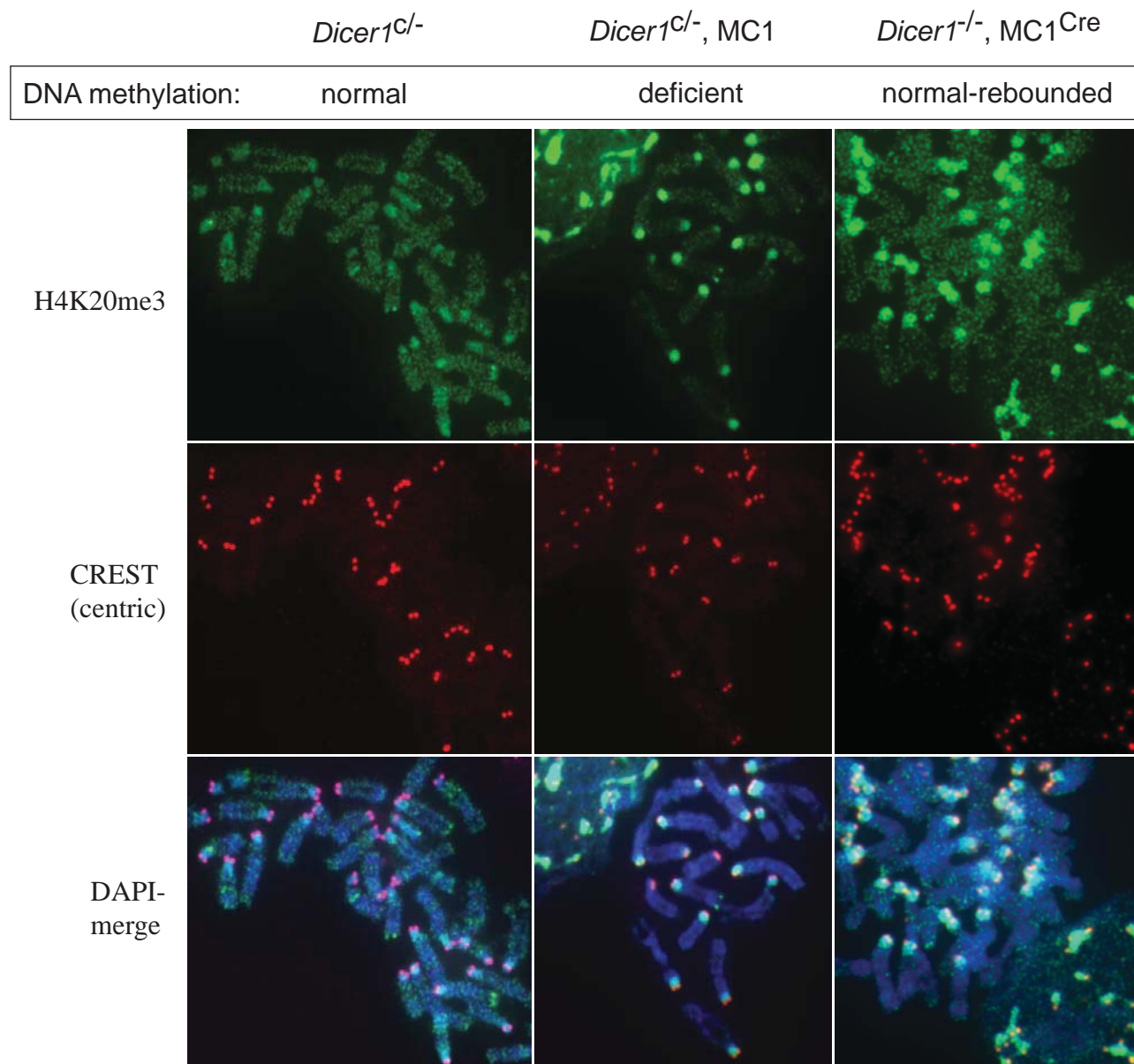

Supplement: Figure S1 — Immunofluorescence for markers of pericentric heterochromatin. (A) Panels: First row; metaphase spreads were probed with an antibody specific for H3K9me3. Second row; CREST antibody is specific for the centromere. Third row; merge of the two images directly above, concomitant with detection of DNA with DAPI, reveals the pericentric localization of H3K9me3. (B) As for A, except that CBX5 (synonym HP1α) is shown to be localized to the pericentric region. (C) As for A, except that H4K20me3 is shown to be localized to the pericentric region. Dicer1 c/− panels at left are the EP parental cell line. (PDF) [file pgen.1002919.s002.pdf]
