# Supplementary material for: Normal DNA Methylation Dynamics in DICER1-Deficient Mouse Embryonic Stem Cells
Source: PLoS Genet. 2012 Sep 6;8(9):e1002919. doi: 10.1371/journal.pgen.1002919 (PMC3435250; doi:10.1371/journal.pgen.1002919)
Supplement: Text S1 — Primers and sequences. Sequences of primers used in qPCR, bisulphite sequencing assays, EpiTYPER assays and stem-loop cloning are shown. For insertion of the shRNA sequence to be driven by the mU6 promoter, each of two pairs of oligos as shown were preannealed, phosphorylated, then combined and ligated into the inverted BsmBI sites (red highlight). This strategy avoided cloning with full-length oligos containing a stem-loop. The functional guide strand (green highlight), which forms one strand of the siRNA as produced by DICER1 processing of the shRNA, is exactly complementary to the Dnmt1 mRNA sequence. The G418 selection cassette was cloned into the unique AscI site in the orientation as indicated (>). To complete the targeting vector, the cassette was excised with XhoI and SalI and cloned into the unique XhoI site in exon 3 of the ∼6 kb Hprt genomic fragment. The transcription start site is indicated by the G. (PDF) [file pgen.1002919.s004.pdf]

Text S1. Primers and sequences

qPCR primers

mRNA: *Rps7*
upper primer: 5′-CCGGCTAGTTCGTGAATTGG-3′
lower primer: 5′-GAATCCTCCTCTGAGCAATGAAG-3′
TaqMan probe: 5′-/TEX615/AAAAGTTCAGTGGCAAGCACGTGG/3IABkFQ/-3′

mRNA: *Alpl*
upper primer: 5′-CCTGCCTTACCAACTCTTTTGTG-3′
lower primer: 5′-CAGGGCATTTTTCAAGGTCTCT-3′,
TaqMan probe: 5′-/Cy5/AGAGAAAGAGAGAGACCCCAGTTACTGGCGA/3IABkFQ/-3′

mRNA: *Dnmt1*
upper primer: 5′-TGGGTGACAGTGTGTACCTTCCT-3′
lower primer: 5′-ATCCTTCTTTGGGCGTTTCA-3′
TaqMan probe: 5′-/HEX/CCGAGGCCTTTACTTTCAACATCA/3IABkFQ/-3′

mRNA: *Dnmt3a*
upper primer: 5′-CCAGCCCTCGGGTCCTAA-3′
lower primer: 5′-ACCCACATGTCGGTGTAAACTTC-3′
TaqMan probe: 5′-/6-FAM/AGCCACCAG/ZEN/AAGAAGAGAAGAATCCTTACA/3IABkFQ/-3′

mRNA: *Dnmt3b*
upper primer: 5′-TGGAGTTCAGTAGGACAGCAAAGT-3′
lower primer: 5′-GTTTTTGCCCTGTCTGATGGA-3′
TaqMan probe: 5′-/HEX/AAAGAAAGTGCAGACAATAACCACCAAGTCGA/3IABkFQ/-3′

mRNA: *Dnmt3l*
upper primer: 5′-GAGTTACTGCACCATCTGCTGTTC-3′
lower primer: 5′-ACTCGAAACAGTAGCATCTGGTACA-3′
TaqMan probe: 5′-/6-FAM/TACCCTGTT/ZEN/CATCTGTGAGAGCCCCG/3IABkFQ/-3′

mRNA: *Rbl2*
upper primer: 5′-TGAGAGCAGAAGCCATCAGAATT-3′
lower primer: 5′-CCTCATCACTGGGCTGGAAT-3′
TaqMan probe: 5′-/6-FAM/TCCAACCGA/ZEN/ACTAAATACAGACAGAGCCAGTAGA/
3IABkFQ/-3′

Bisulphite sequencing primers

Chr 1 subtelomeric region, bases 197167907-197168572 NCBIM37
upper primer: 5′-AAGATGGTGAGTGTAGGTGTTAGTTGAG-3′
lower primer: 5′-TCTTTCTTCTAACTTCTATAACTTCCTATTACCTT-3′
amplicon size: 666 bp
CpGs analysed: 17

Chr 19 subtelomeric region, bases 61333524-61333949 NCBIM37
upper primer: 5′-AGGATTTGTTGATAAGTTTTGGGTTTTTGG-3′
lower primer: 5′-CTAATTCAAAAATAAAAATTTCTCCCCTAC-3′
amplicon size: 426 bp
CpGs analysed: 18

EpiTYPER primers

*Locus*_assay no.: *Xist*_1, Epipanel
upper primer:
5′-AGGAAGAGAGGTAGAGGATTAGGGGATAGGGTTTA-3′
lower primer:
5′-CAGTAATACGACTCACTATAGGGAGAAGGCTCACATTCAAAAACATATACCACCTCT-3′
amplicon size: 319 bp
CpG units analysed: 2, nos. 1/5.6; CpGs not analysed: CG
gene region analysed: exon 1, CpG-rich region 2
sequence:GCAGAGGACTAGGGGATAGGGCTCAGCGTGGGTGTGGGGATTGGCAGGGTGTGTGTGCATA
TGGACCCCTGGCGCGGTCCCCCGTGGCTTTAAGGGCTGCTCAGAAGTCTATAAAATGGCGGCTCGGGGGCT
CCACCCGAGGCTCGACAGCCCAATCTTTGTTCTGGTGTGTAGCAATGGATTATAGGACATTTAGGTCGTAC
AGGAAAAGATGGCGGCTCAAGTTCTTGGTGCGGTATAACGCAAAGGGCTTTGTGTGTCACATGTCAGCTTC
ATGTCTGAGTTAGCCTGGAGAGGTGGCACATGCTCTTGAATGTG

*Locus*_assay no.: *Xist*_2, Epipanel
upper primer:
5′-AGGAAGAGAGTGTTGTGTGAGTGAATTTATGGTTT-3′
lower primer:
5′-CAGTAATACGACTCACTATAGGGAGAAGGCTCTAAACCCTATCCCCTAATCCTCTA-3′
amplicon size: 351 bp
CpG units analysed: 9, nos. 2/3.4.5/6/8/12.13/14.15/16/17/20.21.22
gene region analysed: exon 1, CpG-rich region 2
sequence:TGCTGTGTGAGTGAACCTATGGCTTAGAAAAACGACTTTGCTCTTAAACTGAGTGGGTGTTC
AGGGCGTGGAGAGCCCGCGTCCGCCATTATGGCTTCTGCGTGATACGGCTATTCTCGAGCCAGTTACGCCA
AGAATTAGGACACCGAGGAGCACAGCGGACTGGATAAAAGCAACCAATTGCGCTGCGCTAGCTAAAGGCTT
TCTTTATATGTGCGGGGTTGCGGGATTCGCCTTGATTTGTGGTAGCATTTGCGGGGTTGTGCTAGCCGGAA
GTAGAAAGCCAAGGAGTGCTCGTATTAGTGTGCGGTGTTGCGCGGAAGCCGCAGAGGACTAGGGGATAGGG
CTCAG

*Locus*_assay no.: *Xist*_4, EpiDesigner (antisense)
upper primer:
5′-AGGAAGAGAGTTATAAGGTTTGGTGGTAGGGGAAT-3′
lower primer:
5′-CAGTAATACGACTCACTATAGGGAGAAGGCTTCAAAATTACAATCTTTATAACCACTCC-3′

amplicon size: 196 bp
CpGs analysed: 3, nos. 5/7.8.9/10; CpGs not analysed: CG
gene region analysed: promoter; CpG-rich region 1; exon 1
sequence:CCATAAGGCTTGGTGGTAGGGGAACTAAAAATGTTCCCCCAAAGCTCCTTAGATGGAGAGAA
ACCACGGAAGAACCGCACATCCACGGGAAACGAGCAAACATGGCTGGAGCAAGCCGTTGCACGCCTTTAAC
TGATCCGCGGCGCTGAAGGCGGAGAGACCAGAAGAGGAGTGGCCACAAAGATTGCAATTCTGA

*Locus*_assay no.: *Gja8*_1 (EpiDesigner)
upper primer:
5′-AGGAAGAGAGGAGGGGTTTTTAGTTTTTAATTTTAGG-3′
lower primer:
5′-CAGTAATACGACTCACTATAGGGAGAAGGCTAAACACCCATTATAAACAACCCTTT-3′
amplicon size: 371 bp
CpGs analysed: 2, no. 2/3
gene region analysed: <1 kb upstream of AUG translation start site
sequence:GAGGGGTTTCCAGCTCTCAACCCCAGGAAAAGAAAGCATAAATTAGGACTGCAGAAGTCAGA
GAAACGTGTAGCCCACATCACAGGCAGTATTTCTTAATGGTTTGAGAATCCAACCTATGGGATCGGGTACA
GAACTGCTAGCTAGCATTTAGCTACCTGATCTTAGACCATCTGTTTAGCCTCAAAAGCCCCTGTTTTCTCA
CACATTGAAAGCAAGCTGGAAACAAACAAACACATCTGTGTGTGCGGGAATTGGCTATGCAGCTGTAACTA
CAATAGGCAGGTCCCTACCATCAGATAGCTAAGGTCTAACAGGACTAAGATAAACGAAATGATGCAACATC
AAAGGGCTGTCCATAATGGGTGTCC

*Locus*_assay no.: *Trpc1*_1, EpiDesigner
upper primer:
5′-AGGAAGAGAGTTGGTTTTATTTTGAGTTTTTTGGA-3′
lower primer:
5′-CAGTAATACGACTCACTATAGGGAGAAGGCTACCTCTCCTATTCAAACTACTTTATCCC-3′
amplicon size: 273 bp
CpGs analysed: 1, no. 3; CpGs not analysed: CG
gene region analysed: <1.5 kb upstream of AUG translation start site
CTGGCTTCACCTTGAGCCTCTTGGAAATTTCACCAAACTCATAAACTTGGAAGTGTATTAAACATTACACG
ACAACGGGGAGATCATTTAGAAACAAAAACTGGAGCAACTTGAGATTATCATTTACATCAAATACAGAAAA
CGTTTGAAAAGATGACTTCGACATCAAAGACTTTTAGAGAATATCTAAAGAGAACATTGATTTTTAAGAAT
TAGTAAGTTAAATGTTGAAAGAGAAACTAATGGGACAAAGTAGCCTGAATAGGAGAGGC

*Locus*_assay no.: *H19*_1, Epipanel (antisense)
upper primer:
5′-AGGAAGAGAGTTTGTTTGAGTTAGTTTTTTAGTTTTTTAA-3′
lower primer:
5′-CAGTAATACGACTCACTATAGGGAGAAGGCTACCACTCCAATTAAAAAAAACCC-3′
amplicon size: 198 bp
CpGs analysed: 4, no. 2/4/5.6/7; CpGs not analysed: CG
gene region analysed: promoter
sequence:TCTGCCTGAGCTAGCCCCTCAGTCCTTCAACATTCCTGCCAGACTCCAGATGCCGAGGTGCT
CCTCGGACCCCACGACTCTCCTCCAGCTCTCCCATCTTCCCCAGTTTCCCCGATACCCCCCACCCCCACCC
CCCACCCCCCTCCCACACCCGGTGCTTCGGGCCCTCTAGCCCGGGCTTTTTCTAACTGGAGTGGC

*Locus*_assay no.: *H19*_4, EpiDesigner
upper:
5'-AGGAAGAGAGTGTAAGGAGATTATGTTTTATTTTTGGA-3'
lower:
5'-CAGTAATACGACTCACTATAGGGAGAAGGCTCCCCCTAATAACATTTATAACCCC-3'
amplicon size: 203 bp
CpGs analysed: 3, no. 1/2.3/4.5; CpGs not analysed: CG
gene region analysed: imprinting control region; CTCF binding site
sequence:TGCAAGGAGACCATGCCCTATTCTTGGACGTCTGCTGAATCAGTTGTGGGGTTTATACGCGG
GAGTTGCCGCGTGGTGGCAGCAAAATCGATTGCGCCAAACCTAAAGAGCCCCCCACCCCTGGTATTGGAA
TTCACAAATGGCAATGCTGTGGGTCACCCAAGTTCAGTACCTCAGGGGGGTCACAAATGCCACTAGGGGG

*Locus*_assay no.: *H19*_5, EpiDesigner (antisense)
upper primer:
5'-AGGAAGAGAGTTTATGATTATGGGATTATAGATGGTGA-3'
lower primer:
5'-CAGTAATACGACTCACTATAGGGAGAAGGCTACCTCAAAAAAATCACAAATACCAC-3'
amplicon size: 242 bp
CpGs analysed: 1, no. 4.5.6; CpGs not analysed: CG
gene region analysed: imprinting control region; CTCF binding site
sequence:CCCATGACTATGGGATCATAGATGGTGATAGGGGAGAAAACTCAATCAGTTGCAATCCGTTT
TAGGACTGCGATGTACGAGACTTCACTGCCGCCCGTGCGGCAACCCTGGTCTTTACACACAAAGGATTCTTT
GCAGAGAGTAAGCCGACCTTGTTGATTTGGGAGTCCGAGTCCACGAGGTACCAGCCTAGAAAATGCATGTG
TCCTGCCCCCCTAGTGGCATTTGTGACCCCCCTGAGGT

Primers used to clone the stem-loop into inverted BsmBI sites

5'-TTTGAGTGTGTGAGGGAGAAATTCA AGAGATTTCTCCCTCACACACTCTTTTT....-3'
3'-....TCACACACTCCCTCTTTAAGTTCTCT AAAGAGGGAGTGTGTGAGAAAAAGAG-5'

Sequence of the m*U6*pr cassette with inverted BsmBI sites for stem-loop cloning

CTCGAGATCCGACGCCGCCATCTCTAGGCCCGCGCCGGCCCCCTCGCACAGACTTGTGGGAGAAGCTCGGC
TACTCCCCTGCCCCGGTTAATTTGCATATAATATTTCCTAGTAACTATAGAGGCTTAATGTGCGATAAAAG
ACAGATAATCTGTTCTTTTTAATACTAGCTACATTTTACATGATAGGCTTGGATTTCTATAAGAGATACAA
ATACTAAATTATTATTTTAAAAAACAGCACAAAAGGAAACTCACCCTAACTGTAAAGTAATTGTGTGTTTT
GAGACTATAAATATCCCTTGGAGAAAAGCCTTGTTT**G**TGAGACGACTGCAGTCGTCTCTTCTCGACGGCGC
GCC.*Pgk1*>*sneo*>poly(A).GGCGCGCCGTCGAC
